# Labile iron in cells and body fluids: physiology, pathology, and pharmacology

**DOI:** 10.3389/fphar.2014.00045

**Published:** 2014-03-13

**Authors:** Zvi Ioav Cabantchik

**Affiliations:** Department of Biological Chemistry, Institute of Life Sciences, Hebrew University of JerusalemJerusalem, Israel

**Keywords:** iron, iron metabolism, chelator, siderophore, mitochondria, iron overload, oxidative stress, fluorescence

## Abstract

In living systems iron appears predominantly associated with proteins, but can also be detected in forms referred as labile iron, which denotes the combined redox properties of iron and its amenability to exchange between ligands, including chelators. The labile cell iron (LCI) composition varies with metal concentration and substances with chelating groups but also with pH and the medium redox potential. Although physiologically in the lower μM range, LCI plays a key role in cell iron economy as cross-roads of metabolic pathways. LCI levels are continually regulated by an iron-responsive machinery that balances iron uptake versus deposition into ferritin. However, LCI rises aberrantly in some cell types due to faulty cell utilization pathways or infiltration by pathological iron forms that are found in hemosiderotic plasma. As LCI attains pathological levels, it can catalyze reactive O species (ROS) formation that, at particular threshold, can surpass cellular anti-oxidant capacities and seriously damage its constituents. While in normal plasma and interstitial fluids, virtually all iron is securely carried by circulating transferrin (Tf; that renders iron essentially non-labile), in systemic iron overload (IO), the total plasma iron binding capacity is often surpassed by a massive iron influx from hyperabsorptive gut or from erythrocyte overburdened spleen and/or liver. As plasma Tf approaches iron saturation, labile plasma iron (LPI) emerges in forms that can infiltrate cells by unregulated routes and raise LCI to toxic levels. Despite the limited knowledge available on LPI speciation in different types and degrees of IO, LPI measurements can be and are in fact used for identifying systemic IO and for initiating/adjusting chelation regimens to attain full-day LPI protection. A recent application of labile iron assay is the detection of labile components in intravenous iron formulations *per se* as well as in plasma (LPI) following parenteral iron administration.

## INTRODUCTION

**FIGURE 1:**
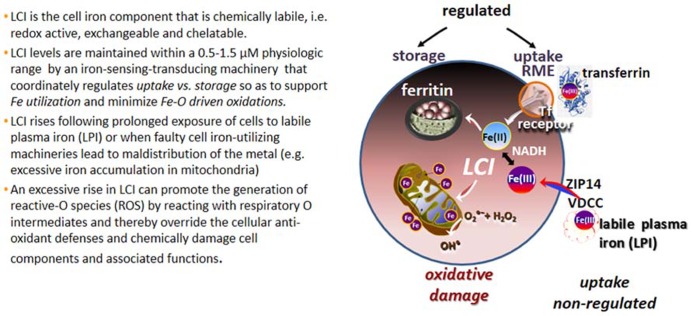
**Labile cell iron (LCI) and labile plasma iron (LPI)**. Proposed routes of labile iron ingress into cells comprise voltage dependent Ca channels (VDCC; Oudit et al., 2006), the Zn transporter ZIP14 (Liuzzi et al., 2006) and others (Sohn et al., 2012).

The various forms of iron present in biological fluids are largely determined by the chemical composition of the medium, particularly its reductive power (commonly dictated by the GSH-NADPH/NADH levels) and the repertoire of substances with metal complexing groups (e.g., carboxylates, phosphates, amides, thiolates, and hydroxylates). In extracellular fluids, essentially all the iron is safely carried by the protein transferrin (Tf) that shields the Fe(III) from the environment and renders it virtually redox-inactive as well as non-exchangeable with, or displaceable by, physiological substances or metals. Thus Tf-bound iron (TBI) is a tightly bound form of ferric iron that by definition is *non-labile* and it remains as such, until conformational changes triggered by binding of Tf to its cognitive Tf receptor (TfR), in conjunction with medium acidification, jointly lead to its release and ensuing reduction to ferrous iron (Crichton, 2001; Cairo and Recalcati, 2007). That complex series of reactions is mechanistically integrated in the physiological process of receptor mediated endocytosis (RME) of TBI that operates in all mammalian cells and serves as the key route of regulated iron uptake (**Figure 1**).

In cellular compartments almost all (>95%) of the iron is also protein-bound, either directly to protein residues or via iron-containing prosthetic groups such as heme or iron sulfur clusters (ISC; Crichton, 2001). Although most of the iron-containing proteins are endowed with catalytic metal centers, for this exposé we consider the redox-active metal as stricto senso labile if it is also physiologically exchangeable and/or pharmacologically chelatable. This fits with the more traditional definition of labile cell iron (LCI) that was introduced in order to describe transitory (i.e., exchangeable) forms of cell iron that are important for cell iron metabolism and homeostasis (Jacobs, 1977; Crichton, 2001). We describe here LCI in physiological and pathophysiological states and its potential relevance to pharmacology and therapeutics. Methodological aspects related to the analysis of LCI and its counterpart in plasma, labile plasma iron (LPI), are the subject of other reviews (Esposito et al., 2002; Kakhlon and Cabantchik, 2002; Petrat et al., 2002; Kruszewski, 2003; Breuer et al., 2007; Brissot et al., 2012).

## LABILE CELL IRON

Many attempts made to chemically define LCI in extracts from lysed cells led biochemists to implicate various Fe^3^^+^ complexes as potential LCI candidates [e.g., nucleotides and even glutathione (GSH); Weaver and Pollack, 1989; Hider and Kong, 2011]. However, those implications should be interpreted with caution as changes in environments (including pH and redox potential) that result from cell de-compartmentalization are likely to result in metal redistribution among potential metal binders. Therefore, in this review we regard LCI as a dynamic parameter that is relevant only for living cells and that prevails under defined conditions, spatial, temporal and environmental (Cabantchik et al., 2009).

### PHYSIOLOGY

The most compelling evidence that LCI prevails in physiological conditions in most mammalian cells, is the demonstrable ability of permeating iron chelators to inhibit the *in situ* (cell or organelle) catalytic contribution of labile iron to basal as well as peroxide-stimulated cell reactive O species (ROS) formation and to inhibit iron-dependent cell functions (Glickstein et al., 2005). LCI is *per se* a generic term used to describe labile iron in the cell as a whole, or in particular cell compartments. The cytosolic and organellar components of LCI are comprised of chelatable complexes of both Fe^2^^+^and Fe^3^^+^, whose relative proportions largely reflect (or are dictated by) the reductive properties and chemical composition of the compartment in question and those in turn depend on cell metabolism (Petrat et al., 2002; Breuer et al., 2007).

The cytosolic LCI, that is “strategically” located at the cross-roads of cell iron metabolism, serves both as metabolic source of metal but also as indicator of cell iron levels. Thus, cells sense and regulate LCI by balancing the uptake of circulating TBI with the storage of unutilized (surplus?) cell iron in shells of ferritin molecules (**Figure 1**). As expected for a dynamic cell parameter, the various LCI pools are likely to vary over time in response to chemical or biological stimuli as well as to metabolic demands/responses. For example, in human K562 or murine erythroleukemia cells, exposure to increasing TBI concentration results in a commensurate rise in cytosolic and mitochondrial LI indicating increased iron uptake by RME and ensuing release into cytosol (and almost concurrent delivery to mitochondria; Shvartsman et al., 2007, 2010; Levi and Rovidac, 2009). Similar results are obtained by modulation of cell ferritin expression (Picard et al., 1998; Kakhlon et al., 2001). Conversely, LCI levels fall in iron starvation, following stable or transient overexpression of cytosolic or mitochondrial ferritin as well as in some mitochondrial disorders that involve aberrant mitochondrial iron accumulation (Rouault, 2006, 2012; Levi and Rovidac, 2009).

The search for putative LCI components have led also to complementary searches for LCI counterparts, namely putative iron chaperons or carriers that, in analogy with copper chaperons, might mediate “safe” iron traffic within cells via formation of non-labile or occluded metal complexes. Accordingly, PBP proteins were implicated as facilitators of iron incorporation into ferritin (Shi et al., 2008) and 2,4 dihydroxybenzoate (2,4-DHB) as universal cytosol-mitochondrial iron chaperon (Devireddy et al., 2010). It must be stressed however, that: (a) neither the putative presence of nM concentrations of 2,4-DHB, seemingly detected in mammalian cells, nor (b) the almost undetectable chelating ability of 2,4-DHB *per se* in buffered medium, let alone (c) in a cellular milieu that is comprised of mM concentrations of organo-phosphates and -carboxylates, could possibly confer upon DHB a putative role as cell iron chaperon, carrier, or the like (Shvartsman and Cabantchik, 2012). For hemoglobin synthesizing reticulocytes, it was hypothesized that iron derived from uptake of TBI by RME is delivered to mitochondria by a mechanism referred as “kiss and run” (Zhang et al., 2005; Sheftel et al., 2007). In that mechanism, vesicle-mitochondria interactions were proposed to provide bridges or channels for trans-vesicular transfer of Fe^2^^+^ to cytosol.

In live-cell fluorescence studies done on human K562 erythroleukemia or murine erythroleukemia (MEL) cells, changes in cytosolic LCI levels occurred in response to increasing concentrations of TBI (but also non-TBI (NTBI) compounds), and almost in parallel to changes in mitochondrial LCI (Shvartsman et al., 2007, 2010). Those studies indicated the operation of various routes of cell iron traffic, most of which are manifested as transient changes in the levels of the cytosolic LCI pool. Observations describing a rapid delivery of iron from the point of entry into cells to mitochondria have also been observed in cells exposed to various NTBI substances (e.g., organic iron salts; Shvartsman et al., 2007). Moreover, in the intestinal CACO-2 cell model, iron trafficked intracellularly from mucosal to serosal cell phases appeared to be shielded in dimetal transporter 1 (DMT1)-containing endocytic vesicles (Núñez et al., 1994; Núñez et al., 1999; Linder et al., 2006). Hitherto, the demonstration of “cell iron passages” as “safe and efficient” routes of iron delivery mediated by cell chaperons or vesicles, awaits experimental support. However, their possible contribution to cell iron physiology does not exclude cellular iron traffic also (or primarily) via LCI pools, whose levels are dynamically monitored live with metal-sensing or redox-sensing probes in response to physiological challenges. 

### PATHOLOGY

It is generally accepted that a major and persistent rise in LCI levels can compromise cell integrity, since excessively accumulated labile iron is prone to engage in the catalytic generation of noxious ROS from reactive O intermediates (ROIs) and those can override the cell antioxidant defenses (**Figure 1**; Kruszewski, 2003). These reactions occur primarily in mitochondria, which not only tend to accumulate excessive LI in various disorders but also suffer the consequences of local ROS formation and ensuing damage. In the various types of systemic siderosis (primary or transfusional), the etiopathology of iron overload (IO) is classically associated with the ability of components of the hemosiderotic plasma to infiltrate cells and raise the LCI levels in both cytosol and mitochondria (Kakhlon and Cabantchik, 2002; Cabantchik et al., 2009). However, a unique feature in inherited mitochondrial disorders caused by faulty biosynthesis/assembly of heme or ISC is the under-utilization of iron that results in mitochondrial iron accumulation/deposition and ensuing cytosolic iron deprivation (Rouault, 2006, 2012; Breuer and Cabantchik, 2009; Camaschella, 2009; Li-Hsuan Huang et al., 2011). That scenario of iron maldistribution (that might be causatively inter-related) is promoted by a vicious circle of increased cell iron uptake induced by a reduction in cytosolic LCI (Li-Hsuan Huang et al., 2011). The latter in turn induces TfR expression via activation of the iron-regulatory IRP-IRE system (Cairo and Recalcati, 2007), leading to increased TfFe uptake and ensuing iron deposition in mitochondria. The resulting phenotypes of regional siderosis are demonstrated in sideroblastic anemia (SA) caused by the mutated gene *alas2* (for XLSA), or *glrx5* (for SA) or *slc25a38 *(for SA) but also in neuro-siderosis caused by the mutated gene fxn (for *FRDA*) or *abcb7 *(for x-linked SA with ataxia, xlsa/a; Rouault, 2006, 2012; Camaschella, 2009; Li-Hsuan Huang et al., 2011).

### PHARMACOLOGY

**FIGURE 2:**
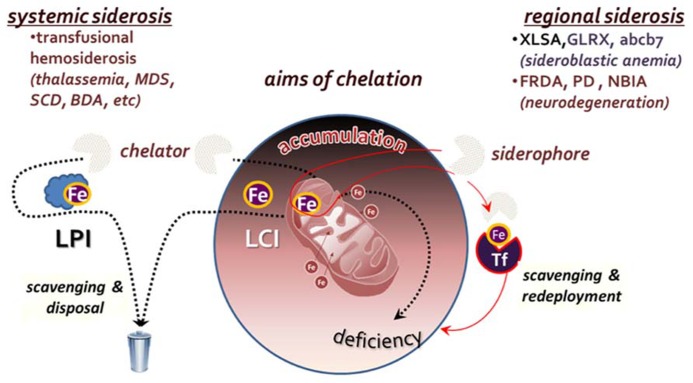
**Aims of chelation therapy for systemic versus regional siderosis**. In **systemic siderosis, **primarily transfusional hemosiderosis (thalassemia major, myelodysplastic syndrome-MDS, sickle cell disease-SCD or Blackfan Diamond anemia-BDA), the major aim is to reduce body iron burden by scavenging and disposing the iron that accumulated in tissues and fluids. In **regional siderosis**, iron accumulation in particular cell compartments is accompanied by a deficiency in another cell compartment (e.g., in various types of sideroblastic anemia or the neurodegenerative disorders Friedreich ataxia-FRDA), or the maldistribution of iron is between different organs. For this cases, chelation is designed to redistribute the accumulated iron within the same cells by direct scavenging and redeployment or via transfer to extracellular transferrin (Tf) and recapture by receptor-mediated endocytosis-RME.

To the extent that a persistently elevated LCI is a risk factor for cell survival (Li-Hsuan Huang et al., 2011; Fleming and Ponka, 2012), metal detoxification by chelation should be regarded as the most direct mode of pharmacological intervention (Hershko et al., 2005; Pietrangelo, 2007; Porter, 2009; Ma et al., 2012; Camaschella, 2013). However, such an approach should be endowed with specificity for labile forms of iron and designed with an adequate regimen so as not to generate long term metal deficiency, local or systemic. In principle, the major goal of chelation is the neutralization or attenuation of iron propensity for catalyzing radical formation. In order for a chelator to meet that goal in an iron overloaded tissue, it must be permeant to cells and endowed with an effective binding affinity for the labile metal (to ensure specificity) and a mode of coordinating complexation that should render the metal essentially non-labile. Those are exemplified by: (a) the tridentate chelator deferasirox (DFR) that binds Fe^3^^+^ with 2:1 stoichiometry and (b) the bidentate deferiprone (DFP) that binds it with 3:1 stoichiometry. As in systemic siderosis, the targets of chelation are both within cells and in extracellular fluids, the goal of chelation is twofold: (a) metal detoxification by means of reducing the iron burden in overloaded tissues and (b) prevention of tissue iron accumulation, by maintaining a low level of LPI, namely the labile components of plasma NTBI (**Figure 2**).

Unlike in systemic siderosis, in regional siderosis, iron is generally maldistributed among cells or cell organelles, often accumulating in some at the expense of iron-sufficient ones. Scavenging of accumulated iron by chelation might accomplish regional detoxification but also concurrently generate deprivation in iron-sufficient or iron-deficient regions (e.g., tissues, cells, or organelles; Breuer and Cabantchik, 2009). Thus, for regional siderosis, treatment should not be limited to scavenging of surplus iron but should be followed by its redeployment, either within or across cells, as depicted in **Figure 2**. Such mode of metal redistribution can be accomplished by chelators with siderophore properties, namely membrane permeant chelators that have a combined accessibility and iron affinity for scavenging LCI in cell and organelles but also the ability to transfer the chelated metal to cell acceptors or the plasma iron acceptor Tf (Kakhlon and Cabantchik, 2002; Breuer and Cabantchik, 2009). A paradigm for that modus operandi is given by DFP, that in addition of scavenging and redeploying iron between cells and extracellular Tf, it has also the ability to correct iron maldistribution *per se* (Sohn et al., 2008, 2011; Breuer and Cabantchik, 2009; Kakhlon et al., 2010; e.g., between mitochondria and cytosol, as found in cells rendered frataxin deficient by shRNA suppression technology) as well as most of the affected cell properties (Kakhlon et al., 2008, 2010).

### DETERMINATION OF LCI IN LIVING CELLS

**FIGURE 3:**
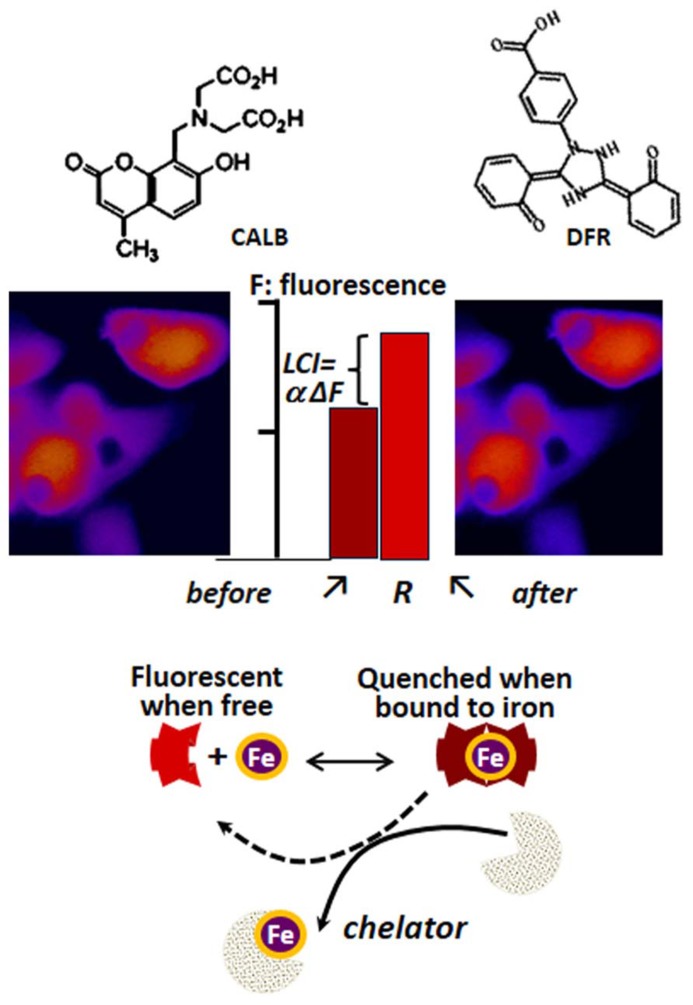
**Principle of labile iron detection with fluorescent metal sensors (FMS) using fluorescence microscopy imaging**. Calcein blue (CALB) as FMS paradigm. The blue fluorescent umbelliferyl iminodiacetic acid binds Fe(III) with a 2:1 or 3:1 stoichiometry and undergoes fluorescence (F) quenching which can be reversed by addition of excess amounts of the chelator deferasirox (DFR). That process, schematically depicted in the lower part of the figure, can be applied to cells (e.g., human hepatic hepG2 cells) by loading them with the acetomethoxy ester (CALB-AM) precursor that generates the fluorescent CALB in the cytosol (fluorescence microscopy imaging, pseudcolor: blue represents relatively low F signal and red high F signal). The addition of an excess amount of a strong permeant chelator (e.g., deferasirox, DFR) leads to a rise in intracellular fluorescence ΔF that denotes the amount of CALB-Fe generated by binding of CALB to LCI. The observed ΔF, which is proportional to LCI, can be converted into actual concentration of iron (Epsztejn et al., 1997; Esposito et al., 2002) that represents the cytosolic LCI pool.

**FIGURE 4:**
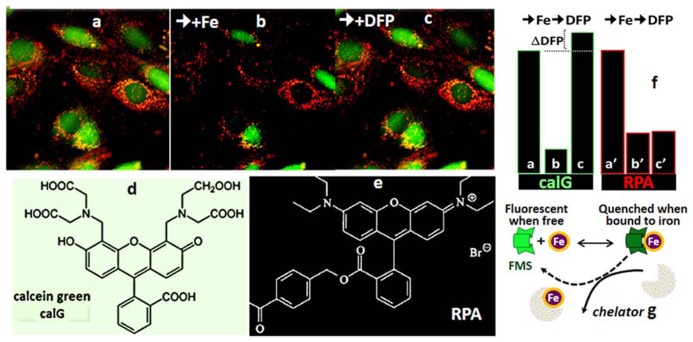
**Calcein green (CALG) and RPA as FMS for simultaneous assessment of Fe ingress into cytosol and mitochondria (fluorescence microscopy imaging)**. The indicated fluorescent FMSs CALG (d) (loaded into the cell cytosol via the CALG-AM precursor) and red RPA (f) (loaded into cell mitochondria by potentiometric distribution) (a) were monitored with time by fluorescence microscopy imaging. Following addition of 5 μM ferric-hydroxyquinoline (1:1) for 10 min (b) the fluorescence intensity (in relative units) in both cytosol and mitochondria dropped substantially (from a to b and a′ to b′ for CALG and RPA, respectively). The reduction in the fluorescence signal elicited by the added Fe provides a measure for the amount of labile metal that gained access to the cytosol in 10 min, until Fe ingress was stopped by addition of 20 μM of the impermeant chelator DTPA-diethylenetriamine pentaacetic acid (to chelate all extracellular Fe). Subsequent addition of 50 μM of the permeant chelator DFP, led to the recovery CALG fluorescence quenched by the added Fe as well the original LCI, denoted by ΔDFP. On the other hand, the quenched RPA signal was not recovered by addition of DFP, due to the relatively poor reversibility of phenanthroline-complexed metal in physiological conditions. g depicts the principle of CALG use as FMS.

The determination of LCI in living cells has been based on spectroscopic probes (herewith referred as fluorescence metal sensors FMS) that detect labile iron *in situ* and in real time by one of the following mechanisms (Petrat et al., 2002; Glickstein et al., 2006; Ma et al., 2006; Breuer et al., 2007): (a) reversible quenching of fluorescence upon binding of iron to a probe endowed with a fluorescent-tagged chelating unit or (b) generation of fluorescence by metal-catalyzed oxidation of a fluorogenic probe (Glickstein et al., 2006; Ma et al., 2006). Changes in fluorescence signal are attributed to labile iron if they are prevented or reversed by a strong and permeant iron chelator that can swiftly gain access to cell compartments and scavenge LCI as well as FMS-chelated iron (!). FMSs like calcein blue (CALB as shown in **Figure 3**) or calcein green (CALG as shown in **Figure 4**) are comprised of one or two metal chelating arms (aminodiacetate or iminodiacetate) linked to fluorescent probes (e.g., fluorescein or methylumbelliferone) that undergo signal quenching upon binding of transition metals like Fe or Cu. The probes can be easily loaded into cells via membrane permeant precursors that are non-fluorescent and non-chelating, e.g., CALG-AM or CALB-AM; AM-acetomethoxy. These probes are hydrolyzed intracellularly, releasing the fluorescent, impermeant chelating CALG (binds iron 1:1) or CALB (binds iron 2:1) that interact (reversibly) with resident labile iron and undergo quenching commensurate with LCI concentrations. That mode of iron sensing is depicted schematically at the bottom of **Figures 3** and **4** for CALB and CALG, respectively, whereby iron added to the free CALB or CALG quenches the FMS and addition of a relatively high concentration of a strong iron chelator restores the quenched fluorescence. In CALB-laden human hepatic HepG2 cells (**Figure 3**), the FMS fills the cytosolic space and binds a fraction of LCI that can be revealed upon addition of excess permeant chelator. Thus addition of DFR elicits a rise in fluorescence ΔF that corresponds to Fe released form the quenched CALB-Fe the chelator (ΔF can be converted to actual concentration of LCI with the aid of appropriate calibration curves as demonstrated earlier for CALG (Epsztejn et al., 1997; Cabantchik et al., 2002; Petrat et al., 2002; Breuer et al., 2007).

**FIGURE 5:**
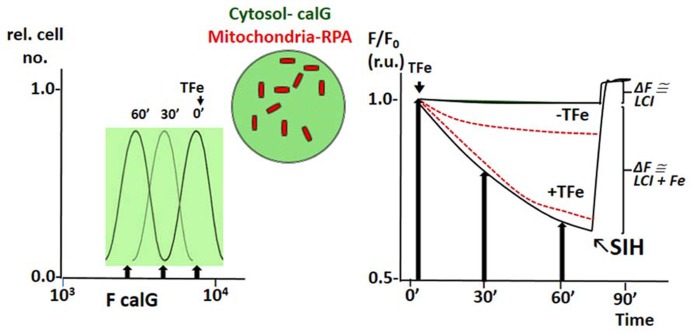
**Example of simultaneous tracing of Fe ingress (from TfFe) into cytosol and mitochondria of human K562 cells by flow cytometry (FC) and front-face fluorescence (plate reader; modified from references** Shvartsman et al., 2010; Shvartsman and Cabantchik, 2012). The fluorescent FMSs calcein green (CALG) and RPA are loaded into K562 cells as described in previous figures, the first in the cytosol and the second in mitochondria (depicted schematically in the center of the figure). Fluorescence intensity (F) is followed with time at different time points by FC or continuously by spectrofluorimetry in a fluorescence plate reader using wavelength settings for fluorescein (CALG) and rhodamine (RPA). The left panel depicts schematically the FC profiles of cell samples taken at different times following addition TfFe (only *F* for green fluorescent calG is shown; arrow indicated median *F* values). The right panel shows values of *F* at different time points (following addition of TfFe or none, as indicated) normalized for values of *F* at *t* time 0 (*F*/*F*_0_) for both CALG (black lines) and RPA (red broken lines) as obtained in a fluorescence plate reader. The arrows indicate the normalized (to time 0) median fluorescence values obtained in the left panel (indicated by arrows). The relatively flat lines represent the mean values in cells not exposed to Tf, to indicate the extent of probe leakage during the measurements. In order to assess the basal LCI level (no TfFe added) and that raised by Fe (from TfFe) after 75′ exposure to TfFe, we obtain a measure of CALG-Fe both in basal conditions (no TfFe added) and at a given time point following addition of TfFe (75′). The measure of CALG-Fe is obtained by addition of a strong permeant iron chelator SIH (salicyl-isonicotinoyl-hydrazone) that scavenges Fe from CALF-Fe raising CALG fluorescence intensity commensurately. The ΔF obtained by addition of SIH provides a measure of LCI.

The principle of LCI measurement demonstrated with the FMS CALB can also be used to assess dynamic changes in various LCI pools with organelle targeted FMS (Glickstein et al., 2005; Shvartsman et al., 2007, 2010; Sohn et al., 2008; Porter, 2009; Cabantchik et al., 2013). **Figure 4** depicts h9c2 cardiac cells double labeled with CALG (cytosol and nucleus) and RPA, a red rhodamine-phenanthroline iron-sensitive probe that targets potentiometrically (binds iron 3:1) to mitochondria. Addition of a permeant iron source reduces the fluorescent intensity of both CALG and RPA (indicating ingress of labile iron to the respective compartment) and subsequent addition of the permeant chelator DFP restores the quenched fluorescence signal of CALG, but not that of RPA (that binds iron tightly and demands extreme conditions for scavenging the bound metal from the phenanthroline:metal 3:1 complex). Parallel time-dependent changes in cytosolic and mitochondrial LCI compartments can be monitored by flow cytometry or fluorescence microscopy as means to assess iron transport into the respective compartments in response to addition of TBI or NTBI (Shvartsman et al., 2007, 2010). As demonstrated in **Figure 5** for TBI, the relative time-dependent changes in fluorescence intensity obtained for either CALG or RPA by flow cytometry match those obtained independently by fluorescence microscopy imaging. **Figure 5** depicts both the resident cytosolic LCI prior to and after addition of TBI (labeled LCI and LCI+, respectively) that are revealed as changes in CALG fluorescence after addition of the permeant tridentate chelator (salicylaldehyde isonicotinoyl hydrazone SIH). When time-dependent profiles are done on probe labeled cells exposed to different substrate concentrations, it is also possible to obtain the kinetic parameters of iron ingress into the respective LCI pools (cytosolic and mitochondrial), by converting ΔF to LCI concentrations, as described elsewhere (Shvartsman et al., 2007, 2010).

Various types of FMS have been applied to cells as sensors for monitoring changes in LCI by following stoichiometry quenching of fluorescence (Rauen et al., 2007; Prus and Fibach, 2008a, b; Ma et al., 2012). Among those are probes based on phenanthroline, deferrioxamine, or hydroxypyridinones chelating moieties and others originally designed for monitoring intracellular Ca. The major shortcoming of most those probes is the difficulty in reversing the quenched signal in physiological conditions and thereby the difficulty in unequivocally assigning the quenched signal to *in situ* LCI.

**FIGURE 6:**
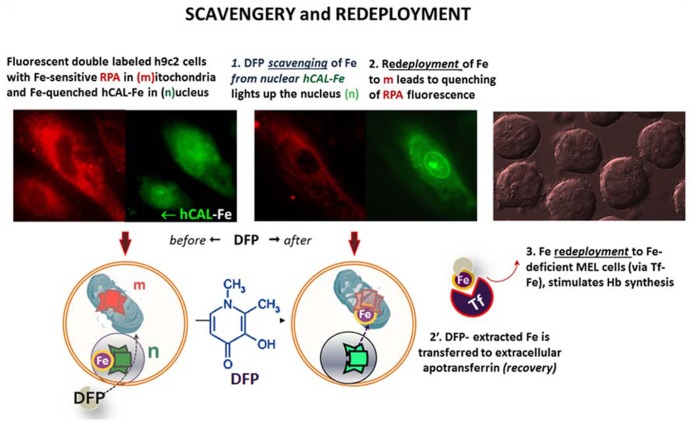
**Intracellular and transcellular redistribution of iron**. The proof of principle of intracellular scavengery and redeployment is depicted schematically in the lower part of the figure, whereas the upper part depicts the experimental results as visualized by fluorescence microscopy imaging. Murine h9c2 cardiomyocytes were double labeled with fluorescent metal sensors: red RPA in mitochondria and green histone-CALG precomplexed to Fe (that partially quenches the probe fluorescence) in the nucleus. Addition of the chelator-siderophore deferiprone (DFP), leads to (1) the scavenging of the Fe from hCAL-Fe, manifested as an increase in nuclear fluorescence and (2) a concomitant quenching of mitochondrial-RPA red fluorescence, that denotes the redeployment of Fe from the intracellular generated DFP-Fe chelate. In addition, DFP-Fe chelates that egress from cells (2′) can transfer the Fe to extracellular apotransferrin (Tf) and the latter (3) in turn, can furnish the metal to Fe-deficient murine erythroleukemia cells (MEL) and thereby support hemoglobinization. All the described steps were experimentally demonstrated in references (Sohn et al., 2008, 2011).

Targeted FMSs have also been used to demonstrate intracellular redistribution of iron (scavengery and redeployment) by addition of the chelator siderophore DFP in a cell model of iron maldistribution (Kakhlon et al., 2008; Sohn et al., 2008, 2011). As depicted in **Figure 6**, h9c2 cells, were pre-labeled with the FMSs RPA (red, for mitochondria) and the fluorescence quenched histone-CALG-Fe (green, for the nucleus) and incubated with the bidentate chelator DFP. As schematized in the lower panel, an increase in nuclear fluorescence elicited by added DFP denotes iron scavengery from the CALG-Fe moiety, whereas a decrease in RPA fluorescence denoted DFP-mediated redeployment of Fe to mitochondria. As some DFP-Fe might also egress from cells into a medium containing apo-Tf, iron can redeployed to Tf, that in turn can also deliver the metal to other cells. This is depicted in the right panel, whereby the medium from the h9c2 cells treated with DFP offered to iron-deficient erythroid MEL cells supported hemoglobin Hb synthesis.

An additional mode of monitoring LCI in living cells is by assessing the ability of labile iron to catalyze oxidation of fluorogenic analogs of dihydrofluorescein or dihydrorhodamine loaded into cells. In the presence of ROIs (spontaneously generated in the cell or prompted by addition of oxidants like H_2_O_2_ or other peroxides) the labile metal converts *in situ* the non-fluorescent dihydro-probes into the respective fluorescent probes. As with the other FMS, the contribution of LCI to the time-dependent generation of fluorescence is assessed by the degree of inhibition attained by addition of a permeant chelator (Glickstein et al., 2006; Ma et al., 2006).

## LPI AND NTBI

### PATHOLOGY AND PHARMACOLOGY

In systemic IO, plasma is the compartment that harbors the pathophysiological source of uncontrolled iron ingress into cells and ensuing tissue IO (Hershko et al., 1978, 2005; Graham et al., 1979; Pietrangelo, 2007). The most implicated component of that source is LPI (Graham et al., 1979), the plasma counterpart of LCI that represents the labile fraction of plasma NTBI (Breuer et al., 2000; Esposito et al., 2003). As LPI comprises the redox-active and exchangeable forms of iron in native plasma that are also direct pharmacological targets of chelation (Pootrakul et al., 2004), it represents a parameter with potential diagnostic as well as therapeutic (i.e., theragnostic) value (Cabantchik et al., 2013). The generic term NTBI was originally introduced to denote the non-physiological, low molecular weight forms of iron that is not tightly associated with Tf and appears in plasma of IO patients (Hershko et al., 1978; Graham et al., 1979). Those forms were postulated on the basis that iron levels in plasma of IO patients often supersede total iron binding capacity (TIBC). Since a substantial fraction of NTBI is apparently adsorbed to plasma proteins, its chemical detection has posed technical difficulties in native plasma/serum unless pretreated with metal mobilizing agents like the polycarboxylate nitrilotriacetate (NTA) or diethylenetriamine pentaacetic acid (DTPA; Hershko et al., 1978; Graham et al., 1979; Singh et al., 1990; Gosriwatana et al., 1999; Kolb et al., 2009). Although several tests have been performed to ascertain that the applied extraction measures do not generally lead to some mobilization of Tf iron, those claims have been challenged leading to the application of more gentle mobilization conditions (Kolb et al., 2009). However, in general, most mobilizing maneuvers applied to plasma/sera that contains any of the chelators used in clinical practice, can lead to mobilization from highly saturated Tf (Kolb et al., 2009). A more serious difficulty pertains to the use of the generic term NTBI to denote plasma iron forms that are unique to IO and presumed to be potentially toxic. Defining “*something that is by what it is not*” (classically known as an *apophasis, from Greek* άπóφασις), can be confusing and in practice oxymoronic. For example, plasma that does not contain pathological species contains in fact NTBI in the form of ferritin or iron-chelates generated during iron chelation therapy or polymeric iron that is given supplementary via parenteral routes and detected in plasma by NTBI assays. On the other hand LPI is a parameter that denotes, operationally, the level of labile iron species in native plasma/serum irrespective of treatment or medium composition, an asset that can also become a liability if plasma/serum is not properly stored or some components of serum that affect its redox properties undergo extreme modifications (Breuer et al., 2012).

Attempts were done to identify the membrane transport mechanisms involved in NTBI uptake into cells that cause IO and oxidative damage. However, few studies took into consideration : (a) the actual repertoire of iron permeant species (i.e., the iron complexes that comprise the iron overloaded plasma containing NTBI) and their relative concentrations at different degrees of IO, (b) the composition of the medium milieu (e.g., the presence of high protein that limits substrate availability and redox-active substances that affect substrate composition) and (c) the different transport agencies present in different cell types. Those agencies comprise a variety of transporters or carriers of metals or metal-complexes, ionic channels or even the means for non-specific adsorptive endocytosis. As most NTBI transport studies have only been done with model simulating substrates in protein-free media and with model cells, their relevance to the etiopathology of tissue iron accumulation in systemic IO remains to be experimentally demonstrated (**Figure 1**). Recent studies indicated dimeric and oligomeric iron-citrate complexes as potential NTBI candidates (Evans et al., 2008), although the chemical speciation is likely to vary with the level of IO and the natural history of the disease in a given patient (Silva and Hider, 2009). Likewise, NTBI association with plasma proteins is exacerbated in oxidative conditions, such as those that prevail under inflammation or in chronic diabetes (Arezes et al., 2013). A single study with T lymphocytes demonstrated that oligomeric ferric citrate is taken up by T lymphocytes (Arezes et al., 2013) and another study indicated that a substantial fraction of iron that accumulated from exposure of cells to NTBI-containing human plasma was by adsorptive endocytosis (Sohn et al., 2012).

### DETERMINATION OF LPI/PLASMA NTBI

**FIGURE 7:**
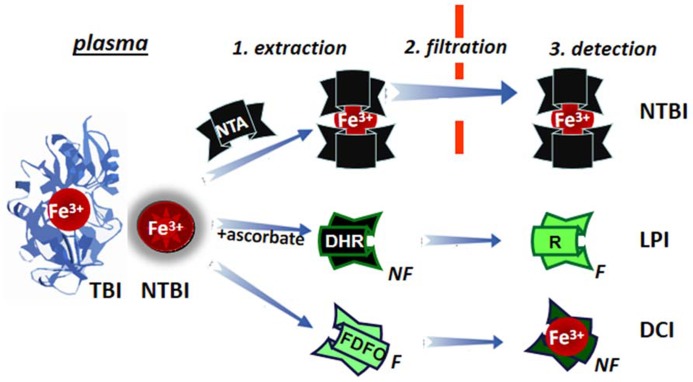
**Principles of determination of LPI and of plasma NTBI**. Plasma/serum non-TBI (transferrin bound iron) indicated as NTBI is measured by the three indicated methodologies. NTBI is measured by its extraction (1) from serum with relatively high (10–80 mM) concentrations of nitrilotriacetic acid (NTA), size filtration (2) and iron detection by colorimetric methods or by addition of iron complexing agents and analysis by HPLC. The labile plasma iron (LPI) component of NTBI is analyzed by exposing native plasma or serum to physiological concentration of ascorbate in the presence of the fluorogenic dihydrorhodamine (DHR), whose time-dependent conversion from non-fluorescent (NF) to green (F) fluorescent 1,2,3, rhodamine (R) is followed in a plate reader (or in a fluorimeter) and the fraction of the signal change inhibited by deferrioxamine (or another strong and specific iron chelator) is attributed to labile iron. Performance of LPI measurements in the presence of 0.1–1.0 mM NTA, provides an equivalent estimate of NTBI (Breuer et al., 2012). The directly chelatable iron (DCI) component of NTBI is assessed with a strong fluorescent (F) chelator such as fluoresceinated-deferrioxamine (FDFO) that upon binding of Fe(III) undergoes stoichiometric quenching to non-fluorescent (NF) chelate FDFO-Fe.

The measurement of a few μM of plasma NTBI in the presence of 70 to >100% saturated TBI (30–50 μM) poses some methodological problems. Two different approaches have tried to overcome them (schematically depicted in **Figure 7**): (1) by extraction of plasma NTBI with iron mobilizing/extraction/chelating agents like NTA or DTPA followed by size filtration (to separate NTBI from TBI) and detection of the iron by iron complex formation and direct measurement of colored complexes or following HPLC analysis (Singh et al., 1990; Gosriwatana et al., 1999; Kolb et al., 2009) and (2) by the detection of LPI in native plasma/serum by: (a) prompting resident LPI (with physiological concentrations of ascorbate) to either catalytically convert the non-fluorescent probe dihydrorhodamine (DHR) into the fluorescent rhodamine (R; Esposito et al., 2003) or co-catalyze with bleomycin chemical changes in chromophoric substrates (Halliwell et al., 1988; Evans and Halliwell, 1994) or (b) binding the labile iron with a fluorescent-chelator that undergoes a commensurate quenching of fluorescence, as exemplified in **Figure 7** with fluoresceinated DFO (FDFO; Breuer et al., 2001). For either modality (a or b) of method 2, a specific iron chelator (DFO or DFP in excess) is added to a parallel sample in order to assess the specific contribution of labile iron to the observed change in fluorescence elicited in the test sample.

The LPI/NTBI assays have been used extensively to monitor chelation efficacy in transfusional siderotic patients treated with different chelators (Cabantchik et al., 2005; Daar et al., 2009; Zanninelli et al., 2009; Greenberg et al., 2010; Danjou et al., 2014) or hemochromatosis patients following phleboptomy (Le Lan et al., 2005), particularly for establishing: (a) the attainment of sufficiently low LPI (<0.1 μM) at trough levels of chelator as a goal of a particular therapeutic regimen (Cabantchik et al., 2005; Zanninelli et al., 2009) an the appearance of LPI as a criterion for initiating chelation therapy in polytransfused children (Danjou et al., 2014). An attempt has also been done to compare different assays for assessing NTBI (not LPI; Jacobs et al., 2005) but a newer completed one that included also LPI should provide more comprehensive and updated information regarding the usefulness of the LPI/NTBI assays in the clinical setting.

## CONCLUSION AND PERSPECTIVES

With presently available methodologies based on FMS, it has become possible to obtain real time measures of LCI and their compartments in both physiological and pathological conditions (i.e., systemic and regional siderosis) and of LPI/NTBI in body fluids as early marker of systemic IO and treatment efficacy. Of particular importance is the ability to assess biological responses to pharmacological measures (e.g., intravenous iron supplementation or iron chelation) and correlate them with clinical outcomes. That area still needs further strengthening, particularly in establishing the thresholds of changes in LPI and LCI that define a given pathological state, either overt or impending.

Unfortunately, hitherto, it has been difficult to define the chemical components of LCI and LPI in biological systems, largely because of their heterogeneous character and variable nature of the media they dwell in. The same pertains to hypothesized iron chaperons that might be operative in mammalian cells and play some physiological role in intracellular iron traffic and metabolism. Although LCI is the parameter closely associated with tissue iron accumulation and ensuing iron-dependent damage, from the clinical perspective, only LPI has thus far proven diagnostically therapeutically relevant for assessing impending/emerging IO and therapeutically, for initiating chelation and monitoring its short term and long term efficacy.

## Conflict of Interest Statement

The author declares that the research was conducted in the absence of any commercial or financial relationships that could be construed as a potential conflict of interest.
